# Biochemical characteristics and synergistic effect of two novel alginate lyases from *Photobacterium* sp. FC615

**DOI:** 10.1186/s13068-019-1600-y

**Published:** 2019-11-04

**Authors:** Danrong Lu, Qingdong Zhang, Shumin Wang, Jingwen Guan, Runmiao Jiao, Naihan Han, Wenjun Han, Fuchuan Li

**Affiliations:** National Glycoengineering Research Center and Shandong Key Laboratory of Carbohydrate Chemistry and Glycobiology, Shandong University, 72 Binhai Rd, Qingdao, 266200 People’s Republic of China

**Keywords:** Alginate, Alginate lyase, Synergistic effect, Biofuel, Oligosaccharide sequencing

## Abstract

**Background:**

Macroalgae and microalgae, as feedstocks for third-generation biofuel, possess competitive strengths in terms of cost, technology and economics. The most important compound in brown macroalgae is alginate, and the synergistic effect of endolytic and exolytic alginate lyases plays a crucial role in the saccharification process of transforming alginate into biofuel. However, there are few studies on the synergistic effect of endolytic and exolytic alginate lyases, especially those from the same bacterial strain.

**Results:**

In this study, the endolytic alginate lyase AlyPB1 and exolytic alginate lyase AlyPB2 were identified from the marine bacterium *Photobacterium* sp. FC615. These two enzymes showed quite different and novel enzymatic properties whereas behaved a strong synergistic effect on the saccharification of alginate. Compared to that when AlyPB2 was used alone, the conversion rate of alginate polysaccharides to unsaturated monosaccharides when AlyPB1 and AlyPB2 acted on alginate together was dramatically increased approximately sevenfold. Furthermore, we found that AlyPB1 and AlyPB2 acted the synergistic effect basing on the complementarity of their substrate degradation patterns, particularly due to their M-/G-preference and substrate-size dependence. In addition, a novel method for sequencing alginate oligosaccharides was developed for the first time by combining the ^1^H NMR spectroscopy and the enzymatic digestion with the exo-lyase AlyPB2, and this method is much simpler than traditional methods based on one- and two-dimensional NMR spectroscopy. Using this strategy, the sequences of the final tetrasaccharide and pentasaccharide product fractions produced by AlyPB1 were easily determined: the tetrasaccharide fractions contained two structures, ΔGMM and ΔMMM, at a molar ratio of 1:3.2, and the pentasaccharide fractions contained four structures, ΔMMMM, ΔMGMM, ΔGMMM, and ΔGGMM, at a molar ratio of ~ 1:1.5:3.5:5.25.

**Conclusions:**

The identification of these two novel alginate lyases provides not only excellent candidate tool-type enzymes for oligosaccharide preparation but also a good model for studying the synergistic digestion and saccharification of alginate in biofuel production. The novel method for oligosaccharide sequencing described in this study will offer a very useful approach for structural and functional studies on alginate.

## Background

Currently, macroalgae and microalgae are attracting increasing attention and are emerging as alternative and environmentally friendly feedstocks for the production of biofuels, such as bio-ethanol, bio-hydrogen and bio-oil, due to rising energy demands [1–4]. Moreover, algae are widely used in the food, cosmetic, and pharmaceutical industries because they can produce some high-value products that are beneficial to human health, such as bio-pigments, vitamins and minerals [5, 6]. As an alternative biomass, macroalgae in particular possess several crucial features: they require no fresh water, arable land, or fertilizer; they can control atmospheric CO_2_; and their cultivation avoids the conflict between food and fuels [7, 8]. The main polysaccharide component of brown macroalgae is alginate, which composes approximately 40% of the dry weight of algal biomass [9]. Alginate is a linear polysaccharide composed of repeating units of β-d-mannuronate (M) and its C5 epimer, α-l-guluronate (G), which are arranged into homopolyuronic blocks (polyM or polyG) and heteropolyuronic blocks (polyMG or polyGM) [10–12]. Alginate is the major component of the brown algal cell wall and has recently attracted interest due to its potential for biofuel, food and pharmaceutical applications [13, 14].

Alginate lyase is a polysaccharide-degrading enzyme that specifically degrades alginate via a β-elimination reaction, forming a 4, 5-unsaturated double bond at the non-reducing ends of products; the unsaturated uronic acid residues are designated as the Δ units [15–17]. Alginate lyases are widespread, and based on their primary structures, they have been classified into seven polysaccharide lyase (PL) families in the Carbohydrate-Active Enzymes (CAZy) database: PL5, PL6, PL7, PL14, PL15, PL17 and PL18 [18–22]. According to their substrate specificity, alginate lyases can be divided into three classes: polyM-specific lyases, polyG-specific lyases and bifunctional lyases. Moreover, according to their substrate degradation pattern, alginate lyases can be distinguished as endolytic and exolytic enzymes. Most characterized alginate lyases belong to the endolytic type and possess relatively high enzyme activity [23]. Endolytic alginate lyases randomly cleave glycoside bonds within alginate chains and generate a series of unsaturated oligosaccharides (≥ UDP2) as final products, which cannot be directly converted into biofuel [24]. By contrast, exolytic alginate lyases act on the non-reducing/reducing ends of alginate polysaccharides or oligosaccharides to release the unsaturated monomer Δ units, which are needed for the saccharification of alginate in the process of biofuel production [25–29].

Compared to endolytic lyases, only a few exolytic alginate lyases have been identified, and all of them have lower activity and poor stability [29]. Endolytic and exolytic alginate lyases often co-exist in the same genome and are thus thought to work synergistically to cause the fast and complete digestion and utilization of alginate by bacteria. However, the synergistic mechanisms of endolytic and exolytic alginate lyases from the same bacterium strain remain to be investigated in detail, although a few studies have attempted to exploit the synergistic effect of endolytic and exolytic alginate lyases to maximize the alginate saccharification rate by optimizing the conditions of the enzymatic reaction [30, 31]. The biochemical characteristics and crystal structures of an endolytic alginate lyase, AlyA1, and an exolytic alginate lyase, AlyA5, from the marine flavobacterium *Zobellia galactanivorans* were studied in detail, but their synergistic effect was not investigated [32]. Although the synergistic effect of two exolytic alginate lyases, Oalc6 and Oalc17, was clearly elucidated based on the complementarity of their substrate specificity, specific activity and stability, their synergistic mechanism with endolytic lyases received little attention [24]. Thus, the synergistic mechanism of endolytic and exolytic alginate lyases still remains to be illustrated.

In this study, two novel alginate lyases, AlyPB1 and AlyPB2, were identified from the draft genome of a marine bacterium, *Photobacterium* sp. FC615 [33]. AlyPB1 and AlyPB2 were characterized as endolytic and exolytic alginate lyases, respectively, and showed a strong synergistic effect on the complete digestion of alginate. Most importantly, we found that the activity of the exolytic lyase AlyPB2 was substrate size dependent, which could reveal the synergistic mechanism of these two enzymes. In addition, using the exolytic lyase AlyPB2 and ^1^H NMR spectroscopy, a novel and simple method for sequencing alginate oligosaccharides was developed for the first time and successfully used to perform structural analysis of the main final products generated by AlyPB1.

## Methods

### Materials and strains

The strains and plasmids used in this study are listed in Table 1. PrimeSTAR™ HS DNA polymerases, restriction endonuclease, and other genetic engineering enzymes were purchased from Takara Inc. (Dalian, China). Cyanoborohydride (NaBH_3_CN), 2-aminobenzamide (2-AB), sodium alginate (alginic acid sodium salt from brown algae, medium viscosity), hyaluronan, chondroitin sulfate, heparin and heparan sulfate were purchased from Sigma-Aldrich. PolyM (*M* > 90%) and polyG (*G* > 90%) were prepared from sodium alginate according to Haug et al. [11, 12]. Saturated alginate pentasaccharide and alginate polysaccharides (10–25 kDa) were obtained by acid hydrolysis of alginate. Unsaturated alginate oligosaccharides (UDP2–UDP10) were prepared by the digestion of alginate using alginate lyase AlyPB1. And all other chemicals and reagents were of the highest quality available.

**Table 1:** Bacterial strains, plasmids, and primers used for sequencing in the present study

Strains and plasmids	Description	Source
Strains		
*Photobacterium* sp. FC615	A GAG-degrading marine bacterium	This study
*E. coli* BL21(DE3)	F-, ompT, hsdSB (rB-, mB-), dcm, gal, λ (DE3), pLysS, Cmr	Novagen
Plasmids		
pET-22b	Expression vector; Ap^r^	Novagen
pET22b-*alyPB1*	pET-22b carrying an amplified NdeI-XhoI fragment encoding the recombinant protein of AlyPB1 or AlyPB2 fused with a His_6_ tag at the C terminus	This study
pET22b-*alyPB2*		
Sequencing primers		
AlyPB1-F	5′-CATATGTCGACCCAAGATACACCAGTACCGG-3′	
AlyPB1-R	5′-CTCGAGGCTCTTCGGTGCAACCTGCAAACG-3′	
AlyPB2-F	5′-CATATGAAGCTGGAGAATGATACTTCAGCA-3′	
AlyPB2-R	5′-CTCGAGCAGCTCGATAGTCACTAACTCGCC-3′	

Restriction enzyme sites are underlined

*Ap*^*r*^ ampicillin-resistant

Marine bacterium *Photobacterium* sp. FC615 [33] was collected from Jiaozhou Bay, near Qingdao city in Shandong province, China. *E. coli* BL21 (DE3) was used for gene expression and it was cultured at 37 °C in Luria–Bertani (LB) broth or on LB broth agar (LB broth supplemented with 1.5% agar) with ampicillin (100 μg/ml).

### Sequence analysis of AlyPB1 and AlyPB2

GC contents (%) of the open reading frames (ORFs) were determined, and the nucleotide sequences of ORFs were translated into corresponding amino acid sequences using Bio-Edit version 7.2.5 [34]. An online similarity search for the protein sequence was performed by the online BLAST algorithm through National Center for Biotechnology Information program. The secretion signal peptides and their types were predicted using the SignalP 4.1 server. Multiple sequence alignments and phylogenetic analyses were performed by MEGA version 5.05 [35, 36]. The theoretical molecular weights (Mw) were calculated through the peptide mass tool on the ExPASy server of the Swiss Institute of Bioinformatics. Protein modules and domains were identified using the Simple Modular Architecture Research Tool (SMART).

### Construction of recombinant expression vectors

The genome sequence of *Photobacterium* sp. FC615 was sequenced and annotated at Meiji Biotech Inc. (Shanghai, China), which contains two putative alginate lyase genes *alyPB1* and *alyPB2*. The genomic DNA was extracted and purified with the commercial genomic DNA purification kit (TianGen Biotech Co. Ltd., Beijing, China). To express AlyPB1 and AlyPB2 in *E. coli* BL21 (DE3), the full-length genes of *alyPB1* and *alyPB2* without the signal peptide sequence were amplified using high fidelity Prime STAR™ HS DNA polymerases (TaKaRa Inc., Dalian, China) and the primer pairs. The primer pairs with restriction enzyme sites were designed according to the inserting site sequences of the expression vector pET-22b (+) (Novagen). Then the gel-recovered PCR products were ligated to the expression vector through the restriction enzyme sites NdeI and XhoI, yielding the recombinant plasmids pET22b-*alyPB1* and pET22b-*alyPB2*. The recombinant expression plasmids pET22b-*alyPB1* and pET22b-*alyPB2* were transformed into *E. coli* BL21 (DE3) cells. The integrities of the nucleotide sequences of recombinant expression plasmids were confirmed through DNA sequencing at Sangon Biotech (Shanghai, China).

### Heterologous expression and purification of AlyPB1 and AlyPB2

*Escherichia coli* BL21 (DE3) cells harboring a recombinant plasmid pET22b-*alyPB1* and pET22b-*alyPB2* were cultured in 100 ml LB medium containing 100 μg/ml ampicillin and shaken for approximately 2 h at 37 °C at 200 rpm. When the OD_600_ of the cell density reached 0.6–0.8, the cells were induced to start target protein expression by addition of the inducer isopropyl 1-thio-β-d-galactoside (IPTG) at a final concentration of 0.05 mM with shaking at 16 °C for 24 h at 220 rpm. The cells were harvested by centrifugation (8000×*g* for 10 min at 4 °C), washed twice using pre-cooled buffer A (50 mM Tris, 150 mM NaCl, pH 8.0), resuspended in buffer A and disrupted by sonication (72 repetitions, 4 s) in an ice cold environment. The cell lysate was centrifuged again (15,000×*g* for 30 min at 4 °C); then, the supernatant fluid of the cell lysate containing the (His)_6_-tagged target protein was loaded onto a column packed with nickel-Sepharose™ 6 Fast Flow (GE Healthcare) equilibrated with buffer A. To prevent the adsorption of impurities, the column was washed with washing buffer (50 mM Tris, 150 mM NaCl, 5 mM imidazole, pH 8.0) before the supernatant was loaded. The target protein was eluted with elution buffer, which was buffer A supplemented with a linear gradient concentration of imidazole (50–250 mM). The purity and molecular weight of the elution fractions were analysed by 13.2% sodium dodecyl sulfate polyacrylamide gel electrophoresis (SDS-PAGE) according to the methods described by Sambrook et al. [37]. The concentration of the target protein was determined using a BCA (bicinchoninic acid) protein assay kit. Finally, imidazole in the elution fractions was removed by ultrafiltration.

### Assay of AlyPB1 and AlyPB2 activities toward various polysaccharide substrates

To determine the substrate specificity of AlyPB1 and AlyPB2, various polysaccharides (e.g., hyaluronan, chondroitin sulfate, heparin, heparan sulfate, alginate, polyM and polyG) were individually dissolved in deionized water to prepare stock solutions (3 mg/ml). The enzymatic reaction system was mixed with 100 μl of 150 mM NaH_2_PO_4_–Na_2_HPO_4_ buffer (pH 8.0), 100 μl of polysaccharide substrates, 30 μl of the appropriately diluted enzyme, and 70 μl of deionized water and then incubated at 30 °C for 12 h. The reactions were terminated by heating in boiling water for 10 min, subsequently cooled to 4 °C and centrifuged at 15,000×*g* for 10 min. The supernatants were collected for analysis. The reaction products were assayed by measuring the increase in absorbance at 235 nm and via gel filtration HPLC.

### Biochemical characterization of the recombinant proteins AlyPB1 and AlyPB2

The optimal pH was determined using the following buffers with different pH values: 50 mM NaAc-HAc buffer (pH 5.0–6.0), 50 mM NaH_2_PO_4_–Na_2_HPO_4_ buffer (pH 6.0–8.0) and 50 mM Tris–HCl buffer (pH 7.0–10.0). These experiments were performed in a total volume of 300 μl at 30 °C for 1 h. The optimal temperature was measured in 50 mM NaH_2_PO_4_–Na_2_HPO_4_ buffer (pH 8.0) at various temperatures, ranging from 0 to 70 °C. To determine the thermostability of the recombinant proteins, their residual enzyme activities were measured after they were incubated at various temperatures from 0 to 70 °C for 0–24 h. The effects of metal ions and chelators were evaluated by measuring the enzyme activities in the presence of various metal ions and chelators, respectively, at concentrations of 1 mM and 10 mM. All reactions were performed in triplicate. After each treatment, the enzyme activity was estimated by measuring the absorbance at 235 nm.

### Enzyme activity assay

The enzymatic reaction system was prepared by mixing 100 μl of 150 mM NaH_2_PO_4_–Na_2_HPO_4_ buffer (pH 8.0); 100 μl of 3 mg/ml sodium alginate, polyG or polyM; 30 μl of the appropriately diluted enzyme; 30 μl of 100 mM β-mercaptoethanol; and 40 μl of deionized water and then allowed to operate under the optimal reaction conditions for 1–10 min. The reactions were terminated by heating in boiling water for 10 min, were subsequently cooled to 4 °C and were centrifuged at 15,000×*g* for 10 min. The supernatants were transferred for analysis as described above. One unit was defined as the amount of enzyme required to release 1 μmol of the reducing sugars/min under the optimal reaction conditions.

### Polysaccharide-degrading properties and oligosaccharide-yielding properties of AlyPB1 and AlyPB2

To determine the enzymatic depolymerization pattern of sodium alginate by AlyPB1 and AlyPB2, enzymatic reactions were initiated and maintained under the optimal reaction conditions for 0–72 h at appropriate time intervals. After incubation, the reaction mixtures were boiled for 10 min, subsequently cooled to 4 °C, and centrifuged at 15,000×*g* for 10 min. The supernatants were collected for analysis. The molar ratio of each unsaturated oligosaccharide fraction in the products was analysed by gel filtration chromatography on a preequilibrated Superdex peptide 10/300 GL column (GE Healthcare) and monitored at 235 nm by a UV detector. The mobile phase was 0.2 M NH_4_HCO_3_, and its flow rate was 0.4 ml/min. Online monitoring and data analysis (e.g., molar ration determination) were performed using the software LCsolution version 1.25.

To investigate the orientation of degradation of AlyPB2, saturated alginate pentasaccharide was fluorescently labeled at the reducing ends using excess 2-AB (Sigma-Aldrich, USA) [38]. 2-AB-labeled saturated alginate pentasaccharide (2-AB-DP5) was digested by AlyPB2 under the optimal conditions, and the degradation products (10 pmol) were removed at appropriate time intervals for time-course experiments. The reaction mixtures were treated as described above and analysed by gel filtration chromatography on a preequilibrated Superdex peptide 10/300GL column (GE Healthcare) using a fluorescence detector with excitation and emission wavelengths of 330 and 420 nm, respectively.

To demonstrate the oligosaccharide-yielding properties of AlyPB1 and AlyPB2, 150 mg of sodium alginate was exhaustively digested with excessive amounts of the enzymes under the corresponding optimal conditions for 72 h. The unsaturated oligosaccharides in the final product were size-fractionized and collected by gel filtration chromatography at 235 nm as described above. The mass/charge ratio of each unsaturated oligosaccharide fraction in the final products was identified by MS on an ion trap TOF hybrid mass spectrometer (LCMS-IT-TOF, Shimadzu, Japan). Electrospray ionization MS analysis was performed in negative ion mode with the following parameters: source voltage, 3.6 kV; nebulizer nitrogen gas flow rate, 1.5 l/min; heat block and curved desolvation line temperatures, 200 °C; and detector voltage, 1.8 kV. The mass acquisition range was set at 200–600.

### Sequencing of unsaturated alginate oligosaccharides using ^1^H NMR spectroscopy combined with digestion by the exolytic lyase AlyPB2

To determine the structures of the main final products produced by AlyPB1, size-defined oligosaccharides (UDP2, UDP3, UDP4 and UDP5) were prepared from the final digest of alginate by AlyPB1 through gel filtration chromatography as described above. The monosaccharide residues located at the reducing ends and next to the Δ units at the non-reducing ends of unsaturated alginate oligosaccharides could be assigned according to their characteristic chemical shift by ^1^H NMR spectroscopy, and thus, the monosaccharide sequences of UDP2 and UDP3 were directly determined by ^1^H NMR spectroscopy as described previously [39–42]. However, the internal monosaccharide residues of larger unsaturated oligosaccharides were difficult to determine using only ^1^H NMR spectroscopy. Thus, UDP4 or UDP5 was partially degraded by the exolytic alginate lyase AlyPB2 to prepare a series of intermediate oligosaccharide products. The intermediate products, i.e. UDP3 from UDP4 (UDP3–UDP4) or UDP3 and UDP4 from UDP5 (UDP3–UDP5, UDP4–UDP5), were size-fractionized and collected by gel filtration chromatography at 235 nm. Next, the structures of these intermediate unsaturated oligosaccharides from UDP4 and UDP5 were determined by ^1^H NMR spectroscopy (Fig. 7). According to the results of the ^1^H NMR spectroscopy analysis of the intermediate products, the internal structure of UDP4 or UDP5 was able to be determined. ^1^H-NMR spectroscopy was performed on a JNM-ECP600 (JEOL, Japan) instrument set at 600 MHz. Each sample (2 mg) was dissolved in 0.5 ml of D_2_O in a 5 mm NMR tube.

### Activity assay of AlyPB2 towards alginate substrates with different molecular sizes

To determine the activity of AlyPB2 towards alginate substrates with different molecular sizes, unsaturated oligosaccharides (UDP2–UDP10) and saturated alginate polysaccharides (10–25 kDa) were prepared from the degradation of alginate by the endolytic lyase AlyPB1 and acid hydrolysis, respectively. The enzymatic reaction system was prepared by mixing 30 μl of 150 mM NaH_2_PO_4_–Na_2_HPO_4_ buffer (pH 8.0), 30 μl of polysaccharide or oligosaccharide substrates (3 mg/ml) with different molecular sizes, 9 μl of the appropriately diluted enzyme, 9 μl of 100 mM β-mercaptoethanol and 12 μl of deionized water and allowed to proceed under the optimal reaction conditions for 1–10 min. The reaction products were treated as described above and assayed by measuring the increase in absorbance at 235 nm.

### Analysis of the synergistic effect of AlyPB1 and AlyPB2

To investigate the synergistic effect of AlyPB1 and AlyPB2, the enzymatic reaction system was prepared by mixing 10 μl of 150 mM NaH_2_PO_4_–Na_2_HPO_4_ buffer (pH 8.0), 10 μl of sodium alginate (3 mg/ml), 1.14 μl (50 mU) of exolytic AlyPB2, 2.7 μl (50 mU) of endolytic AlyPB1 and 6.16 μl of deionized water and allowed to proceed at 20 °C for 10 min. Two control groups containing 100 mU of the endolytic alginate lyase AlyPB1 or 100 mU of the exolytic alginate lyase AlyPB2 were also set up. The resultant products were treated and analysed by gel filtration as described above at a wavelength of 235 nm.

## Results

### Sequence analysis of AlyPB1 and AlyPB2

The genome of the marine bacterium *Photobacterium* sp. FC615 contains two putative alginate lyase genes: *alyPB1* (ORF 02243, GenBank™ Accession No. MN116685) and *alyPB2* (ORF 02710, GenBank™ Accession No. MN116686). An additional ORF, 02280, has been annotated as an alginate lyase gene, but the protein encoded by this putative gene has no enzyme activity on any alginate associated substrates.

**Fig. 1:**
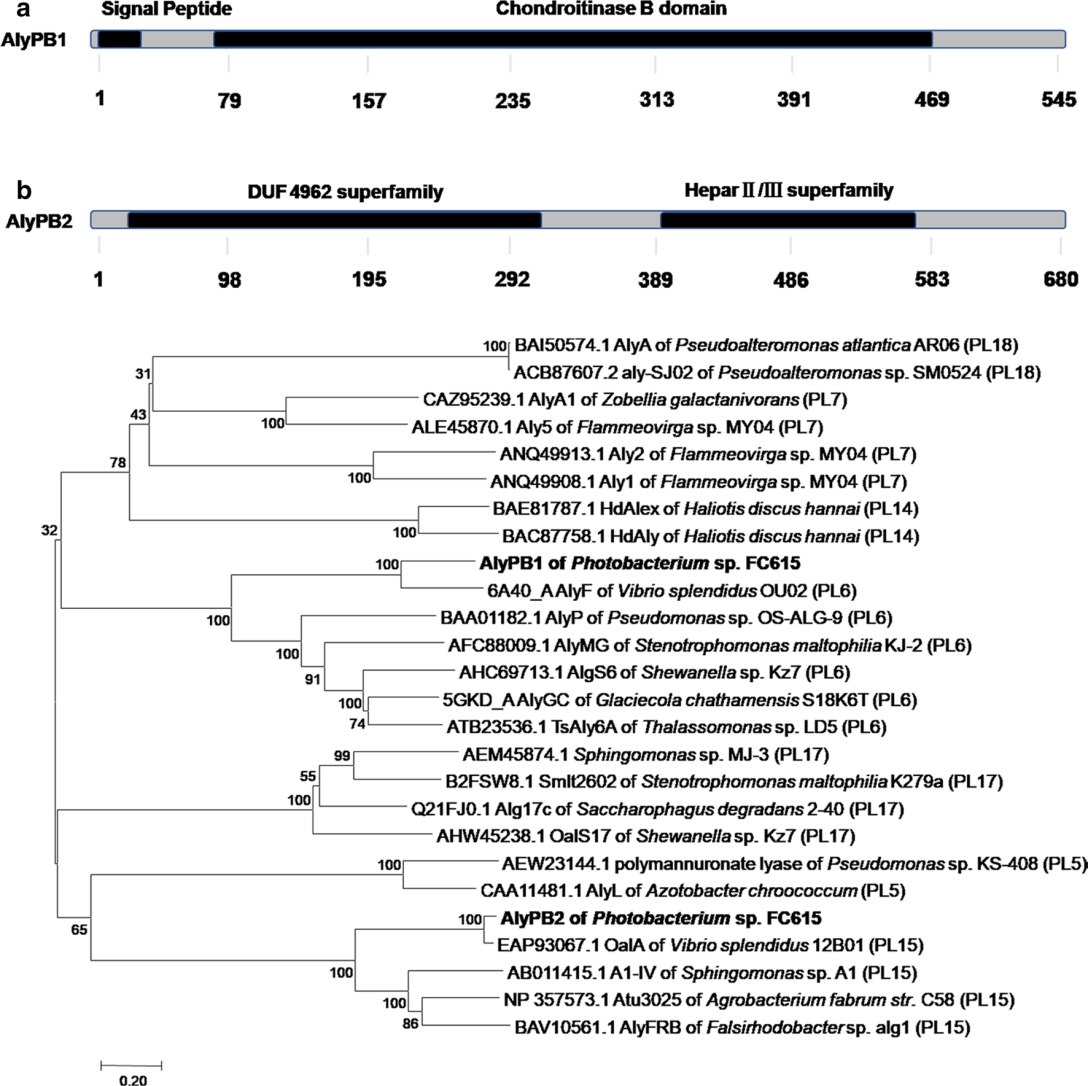
Sequence properties of alginate lyases AlyPB1 and AlyPB2 from *Photobacterium* sp. FC615. **a** Module organization of the alginate lyases AlyPB1 and AlyPB2. AlyPB1 contains a putative N-terminal signal peptide (Met^1^-Ala^21^) and a chondroitinase B domain (Thr^74^-Asp^470^). AlyPB2 contains an unknown function module DUF4962 (Phe^29^-Gln^320^) at N-terminal and a heparinase II/III-like superfamily module (Pro^396^-His^571^) at C-terminal. **b** Phylogenetic analysis of alginate lyases AlyPB1 and AlyPB2 based on protein sequence alignments with reported alginate lyases in various polysaccharide lyase families. The phylogenetic tree was constructed using MEGA version 5.05 software via the neighbor-joining algorithm and associated taxa clustered together in the bootstrap test of 1000 replicates

The ORF of gene *alyPB1* is 1638 bp in length, has a GC content of 51% and encodes a protein, AlyPB1, composed of 545 amino acids including a signal peptide of 21 amino acids. The recombinant protein AlyPB1 composed of 524 amino acids has a calculated molecular mass of 57.6 kDa and an isoelectric point (pI) of 4.88. According to multiple sequence alignments and phylogenetic analysis with characterized sequences in the GenBank database, AlyPB1 belongs to PL6 family (Fig. 1b). AlyPB1 has a chondroitinase B module that is composed of 397 amino acids from Thr^74^ to Asp^470^, but AlyPB1 has no enzyme activity on chondroitin sulfate B (dermatan sulfate), as shown in Fig. 1a. A BLASTp search indicated that among the identified alginate lyases in PL6 family, AlyPB1 shares the greatest identity (60.23%, query cover 97%) with AlyF (6A40_A) from *Vibrio* OU02 [43]; 31.6% identity (query cover 68%) with AlgS6 (AHC69713.1) from *Shewanella* sp. Kz7 [44]; 28.1% identity (query cover 80%) with a poly MG-specific alginate lyase, AlyMG (AFC88009.1), from *Stenotrophomonas maltophilia* [21]; 29.7% identity (query cover 63%) with AlyGC (5GKD_A) from the marine bacterium *Glaciecola chathamensis* S18K6^T^ [18]; 30.6% identity (query cover 57%) with a polyM-specific alginate lyase, AlyP (BAA01182.1), from *Pseudomonas* sp. OS-ALG-9 [10]; and 28.8% identity (query cover 63%) with TsAly6A (ATB23536.1) from *Thalassomonas* sp. LD5 [45].

The ORF of gene *alyPB2* is 2043 bp in length, has a GC content of 49%, and encodes a protein, AlyPB2, composed of 680 amino acids without signal peptide. The recombinant protein AlyPB2 has a calculated molecular mass of 77.7 kDa and an isoelectric point (pI) of 5.01. In the neighbour-joining phylogenetic tree, AlyPB2 was clustered with PL15 family together with the elucidated exolytic alginate lyases Atu3025 (NP_357573.1) from *Agrobacterium tumefaciens* strain C58 [27, 46], A1-IV (AB011415.1) from *Sphingomonas* sp. A1 [25, 26], OalA (EAP93067.1) from *Vibrio splendidus* 12B01 [28] and AlyFRB (BAV10561.1) from *Falsirhodobacter* sp. alg1 [47] (Fig. 1b). According to their primary structure, exolytic alginate lyases are generally separated into PL15 and PL17 family. To date, only five members of the PL15 family have been identified, including AlyPB2. SMART analysis demonstrated that the exolytic alginate lyases in PL15 family contain a DUF4962 superfamily module at their N-terminus and a heparinase II/III superfamily module at their C-terminus. The function of the DUF4962 superfamily domain is unknown. The heparinase II/III domain in AlyPB2 contains 176 amino acids, from Pro^396^ to His^571^, but as found in other alginate lyases, AlyPB2 did not show any heparinase activity (Fig. 1a). A BLASTp search showed that AlyPB2 shares the highest identity with OalA (93.4%, query cover 100%), as well as 42.1% sequence identity (query cover 89%) with Atu3025 and 39.9% (query cover 89%) and 38.1% (query cover 92%) sequence identity with A1-IV and AlyFRB, respectively.

### Heterologous expression of AlyPB1 and AlyPB2 in *E. coli*

**Fig. 2:**
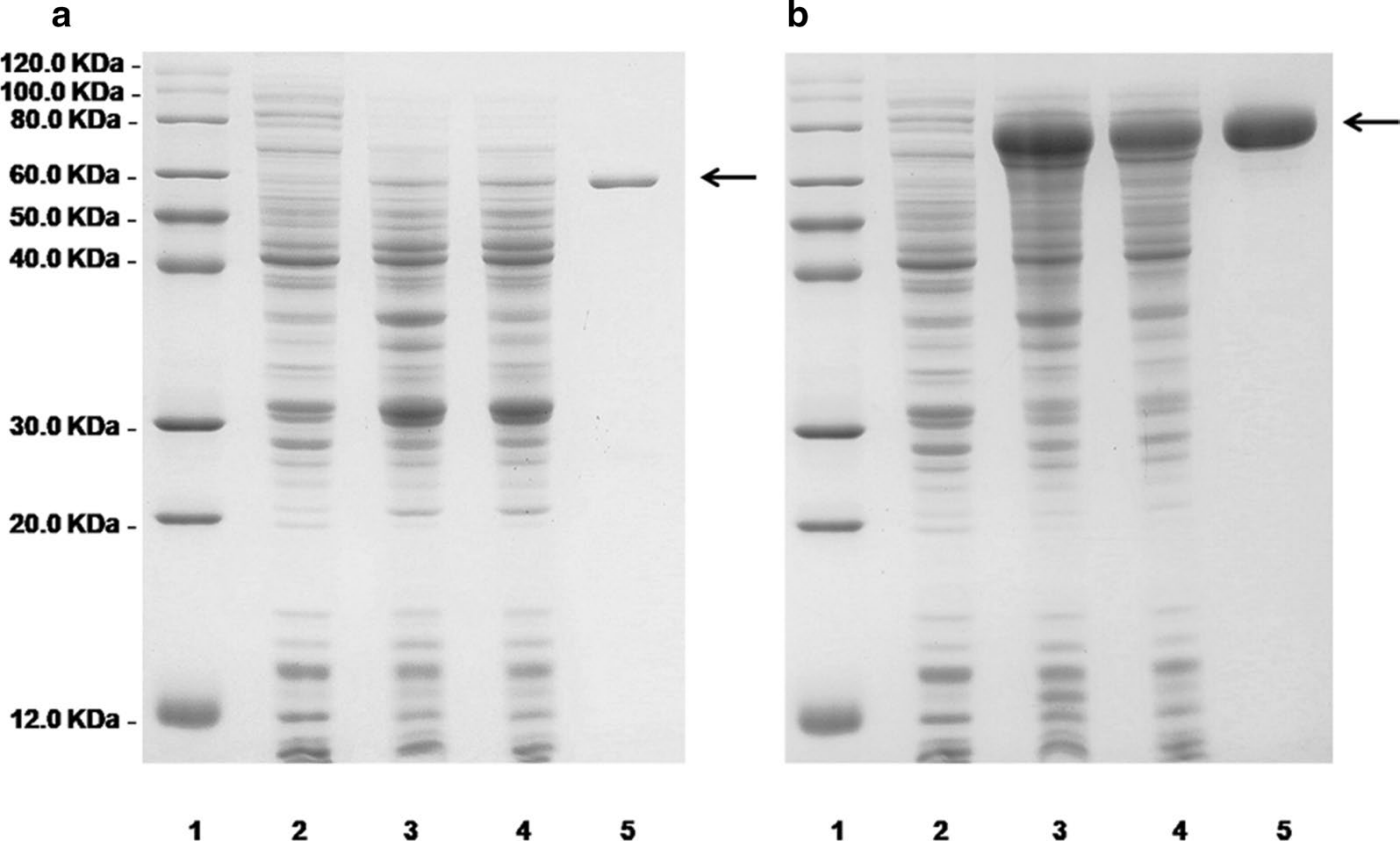
Purification of recombinant AlyPB1 (**a**) and AlyPB2 (**b**) from *E. coli* by Ni^2+^ chelation chromatography. Enzyme purity following each fractionation step was assessed by SDS-PAGE using 13.2% (w/v) polyacrylamide gels, followed by staining with Coomassie Brilliant Blue. Lane 1, unstained protein molecular weight marker ProteinRuler^®^ II (TransGen Biotech); Lane 2, induced cell lysate of *E. coli* cells harboring the control plasmid pET-22b (+); Lane 3, induced cell lysate of *E. coli* cells containing plasmid of pET22b-*alyPB1* (**a**) or pET22b-*alyPB2* (**b**); Lane 4, supernatant fluid of the induced cell lysate; Lane 5, purified recombinant AlyPB1 (**a**) or AlyPB2 (**b**)

Heterologous expression systems for the recombinant alginate lyases AlyPB1 and AlyPB2 were constructed in *E. coli* BL21 (DE3) cells as follows: the *alyPB1* and *alyPB2* were directly amplified from genomic DNA of *Photobacterium* sp. FC615; then, the PCR products were cloned into the pET-22b (+) expression vector downstream of a T7 promoter. The pET-22b (+) expression vector was designed to express recombinant protein labeled with a His_6_ tag at the C terminus. SDS-PAGE showed that AlyPB1 (Fig. 2a) and AlyPB2 (Fig. 2b) purified as a single band at approximately 58 kDa and 78 kDa, respectively, consistent with the calculated molecular masses. The purities of both of the two purified proteins were greater than 98%. The concentration (0.2 mg/ml) of purified protein AlyPB1 was much lower than that (4 mg/ml) of the purified protein AlyPB2.

### Enzymatic characteristics of AlyPB1 and AlyPB2

Seven structurally different polysaccharides, namely, hyaluronan, chondroitin sulfate, heparin, heparan sulfate, alginate, polyM and polyG, were used to investigate the substrate preference of AlyPB1 and AlyPB2 by measuring the increase in absorbance at 235 nm. AlyPB1 could efficiently digest only alginate and polyG, whereas AlyPB2 could efficiently digest alginate, polyM and polyG, suggesting that AlyPB1 and AlyPB2 are alginate lyases that can degrade alginate and associated substrates by a β-elimination mechanism.

**Fig. 3:**
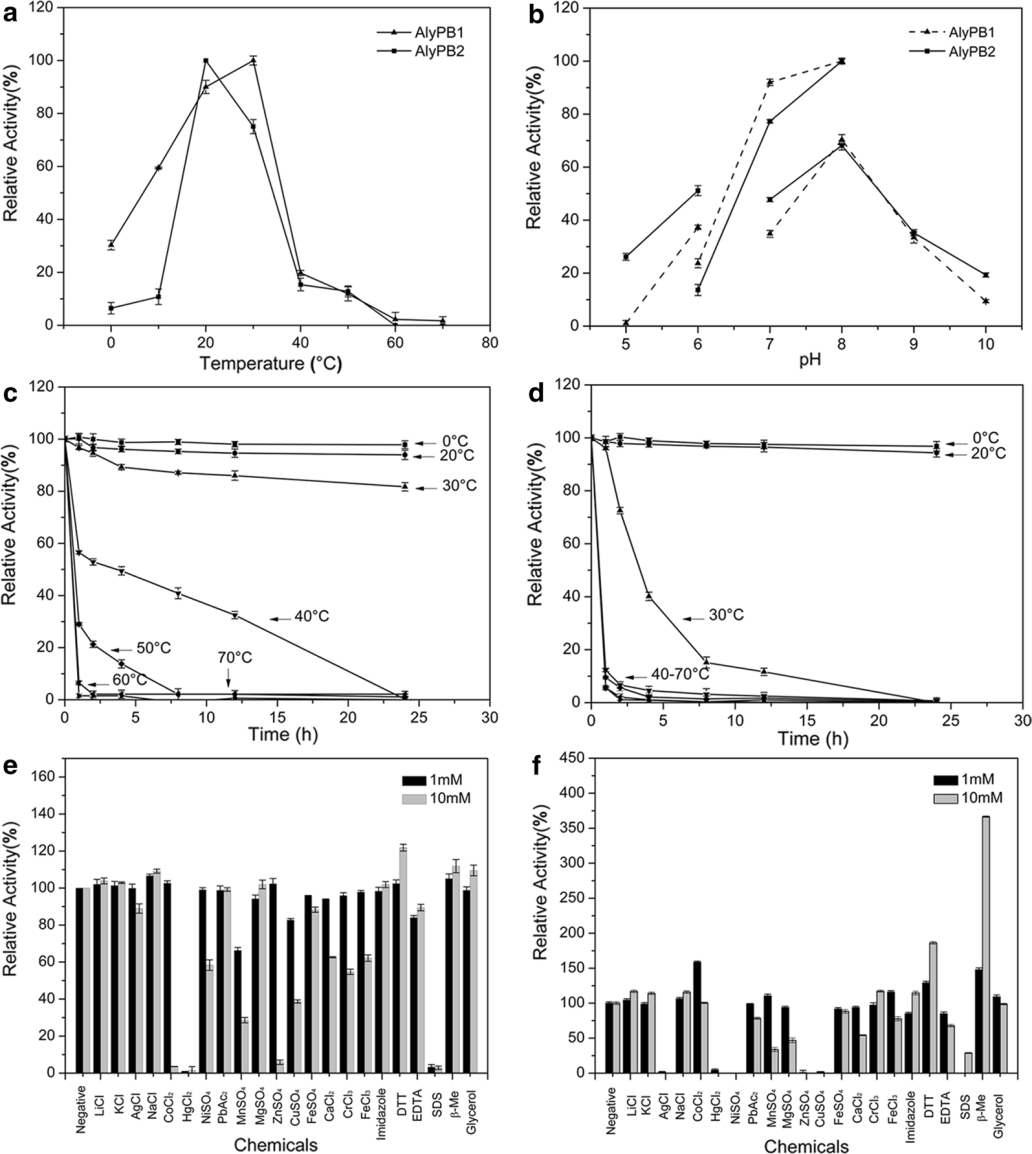
Biochemical characteristics of the recombinant alginate lyases AlyPB1 and AlyPB2. **a** Effects of temperature. The enzyme activities of AlyPB1 and AlyPB2 were each measured using sodium alginate as substrate in the 50 mM NaH_2_PO_4_–Na_2_HPO_4_ buffer (pH 8.0) at various temperatures (0–70 °C) for 1 h. Data are shown as the percentage of the activity of that obtained at 30 °C for AlyPB1 and at 20 °C for AlyPB2 (100%). **b** Effects of pH values. The enzyme activities of AlyPB1 and AlyPB2 against sodium alginate were individually measured in buffers with varying pH values from 5.0 to 10.0 for 1 h at 30 °C of AlyPB1 and at 20 °C of AlyPB2. Data are shown as the percentage of the activity of that obtained in 50 mM NaH_2_PO_4_–Na_2_HPO_4_ buffer (pH 8.0) (100%). **c**, **d** Thermostability of AlyPB1 and AlyPB2. The enzymes were preincubated for 0–24 h under temperatures ranging from 0 to 70 °C, and the residual activities against sodium alginate were estimated at 30 °C of AlyPB1 and at 20 °C of AlyPB2. Data are shown as the activity relative to that of untreated AlyPB1 and AlyPB2. **e**, **f** Effects of various compounds on the enzyme activity of AlyPB1 and AlyPB2. The enzyme activities of AlyPB1 and AlyPB2 against sodium alginate were individually measured in the NaH_2_PO_4_–Na_2_HPO_4_ buffer (pH 8.0) containing a 1 mM or 10 mM concentration of various compounds for 1 h at 30 °C of AlyPB1 and at 20 °C of AlyPB2. Data are shown as the percentage of the activity of obtained in the buffer without tested compounds. Error bars represent mean values of triplicates ± SD

The enzymatic characteristics of AlyPB1 and AlyPB2 were further determined. AlyPB1 showed the highest enzyme activity at 30 °C (Fig. 3a) and retained more than 80% of its original activity after incubation at temperatures from 0 to 30 °C for 24 h (Fig. 3c). The optimal pH of AlyPB1, determined at 30 °C in 50 mM NaH_2_PO_4_–Na_2_HPO_4_ buffer, was 8.0 (Fig. 3b). By contrast, AlyPB2 had a lower optimal temperature (20 °C) and thermostability than AlyPB1 (Fig. 3a, d), though the optimal buffer of AlyPB2 (NaH_2_PO_4_–Na_2_HPO_4_ buffer, pH 8.0) was the same as that of AlyPB1 (Fig. 3b). Furthermore, the activities of both AlyPB1 and AlyPB2 were strongly inhibited by Hg^2+^, Ni^2+^, Mn^2+^, Zn^2+^, Cu^2+^, and SDS (Fig. 3e, f). In addition, AlyPB2 was strongly inhibited by Ag^+^ and Mg^2+^, but AlyPB1 was not (Fig. 3f). Notably, the enzymatic activity of AlyPB2 was increased to 158%, 186% and 366% by stimulation with Co^2+^, DTT and β-mercaptoethanol, respectively (Fig. 3f). By contrast, the activity of AlyPB1 was not significantly stimulated by these chemicals and was strongly inhibited by 10 mM Co^2+^ (Fig. 3e).

Under the optimal conditions of 30 °C in 50 mM NaH_2_PO_4_–Na_2_HPO_4_ (pH 8.0), AlyPB1 showed much higher activity towards polyG (1295 U/mg) than alginate (185 U/mg), whereas AlyPB1 showed extremely low activity towards polyM (< 1 U/mg). By contrast, the specific activities of AlyPB2 towards sodium alginate, polyM and polyG were 10.9, 14.6, and 8.5 U/mg protein, respectively, under the optimal conditions (20 °C in 50 mM NaH_2_PO_4_–Na_2_HPO_4_, pH 8.0). Interestingly, AlyPB1 and AlyPB2 have distinct preferences for polyG and polyM.

### Polysaccharide degradation patterns and oligosaccharide-yielding properties of AlyPB1 and AlyPB2

**Fig. 4:**
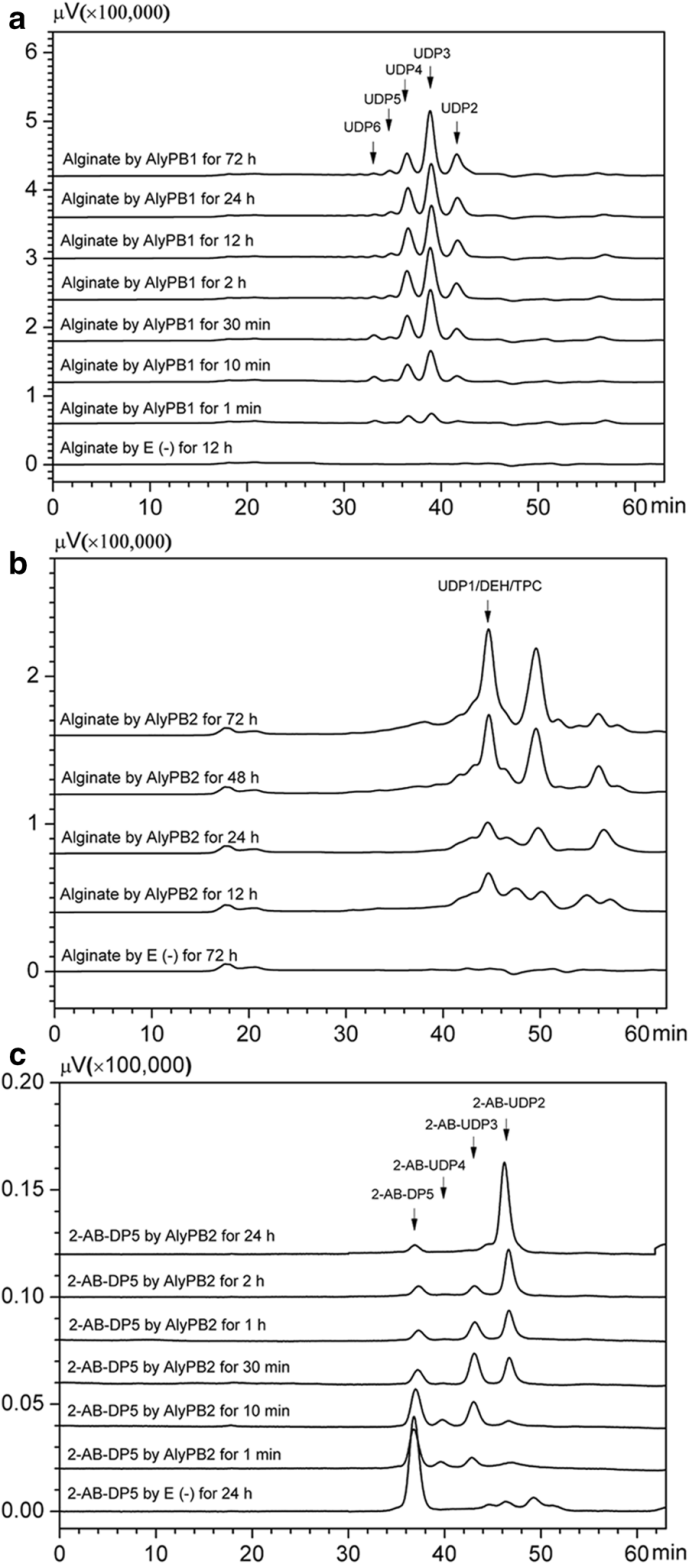
Degradation patterns of alginate polysaccharide by AlyPB1 and AlyPB2. **a** Time-course treatment of sodium alginate (1.0 mg/ml) using AlyPB1 at 30 °C. **b** Time-course treatment of sodium alginate (1.0 mg/ml) using AlyPB2 at 20 °C. The unsaturated oligosaccharide products were analysed through a pre-equilibrated Superdex peptide 10/300 GL column monitored using a UV detector at 235 nm. Degrees of polymerization of oligosaccharides are indicated on the peaks: UDP1, UDP2, UDP3, UDP4, UDP5 and UDP6 represent unsaturated monosaccharide, disaccharide, trisaccharide, tetrasaccharide, pentasaccharide and hexasaccharide; DEH and TPC are both converted products of unsaturated monosaccharide. **c** Analysis for the degradation orientation of exo-lyase AlyPB2. Time-course treatment of 2-AB-labeled saturated alginate pentasaccharide (2-AB-DP5) (10 pmol) with AlyPB2 at 20 °C. The resulting products were analysed by gel filtration as described above but using a fluorescent detector with an excitation wavelength of 330 nm and an emission wavelength of 420 nm. 2-AB-UDP2, 2-AB-UDP3 and 2-AB-UDP4 represent 2-AB-labeled unsaturated alginate disaccharide, trisaccharide and tetrasaccharide. E (−), control treated with inactivated enzyme

Based on their substrate degradation patterns, alginate lyases are mainly classified into three types: endolytic lyases, exolytic lyases and combinations of both [1]. To investigate the substrate degradation patterns of AlyPB1 and AlyPB2, alginate polysaccharide was digested for 0–72 h, and the resultant products were removed at appropriate time intervals and analysed by gel filtration. Unsaturated oligosaccharides with a high degree of polymerization (DP) were the main products in the initial stage of the reaction and were then gradually converted into smaller oligomers (Fig. 4a), indicating that AlyPB1 is an endo-type alginate lyase. Unlike AlyPB1, AlyPB2 did not digest alginate into oligosaccharides with DP ≥ 2 but rather produced unsaturated monosaccharides at all time points (Fig. 4b), suggesting that AlyPB2 is an exo-type alginate lyase that has the ability to remove monosaccharides from the end of alginate chains.

To exactly determine the substrate degradation orientation of the exo-lyase AlyPB2, saturated alginate pentasaccharide (DP5) was labeled with 2-AB at the reducing end and further digested with AlyPB2 in a time-course experiment. The results of the gel filtration assay showed that the digestion of 2-AB-DP5 by AlyPB2 yielded a series of 2-AB-labeled oligosaccharide products with high molecular masses at the beginning of the reaction, and then, the larger products 2-AB-UDP4 and 2-AB-UDP3 were gradually converted into the final product, 2-AB-UDP2 (Fig. 4c). This substrate degradation pattern clearly demonstrates that AlyPB2 is an exolytic lyase that removes uronic acid from the non-reducing ends of alginate chains. Notably, the digestion of 2-AB-labeled alginate oligosaccharides by AlyPB2 finally produced 2-AB-labeled disaccharides rather than monosaccharides due to the steric hindrance effect of the fluorescent label inhibiting further cleavage of 2-AB-labeled disaccharides at the reducing ends of alginate chains.

**Fig. 5:**
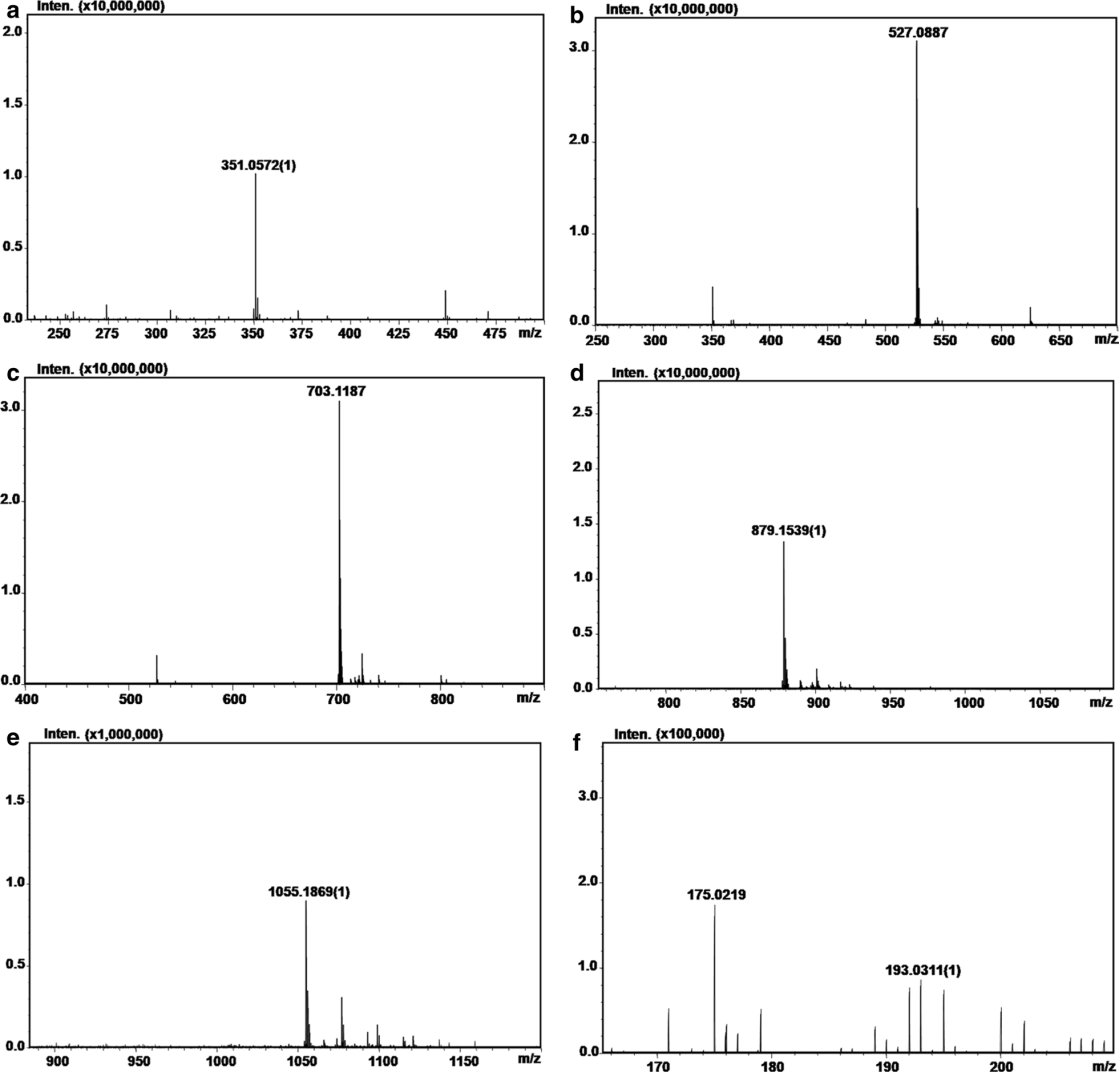
Time-of-flight mass spectra of the final products of alginate digested by AlyPB1 and AlyPB2. The final products obtained from sodium alginate digested by AlyPB1 (**a**–**e**) and AlyPB2 (**f**) were identified by electrospray ionization MS on anion trap TOF hybrid mass spectrometer as described under “Methods”. **a**–**e** The final products UDP2–UDP6 produced by AlyPB1; **f** unsaturated monosaccharide UDP1 and its conversion products DEH and TPC produced by AlyPB2

To further confirm the final products of alginate digestion by AlyPB1 and AlyPB2, alginate polysaccharide was digested with an excess of each enzyme under the optimal conditions for 72 h. The final products were size-fractionated and collected by gel filtration chromatography with detection at 235 nm. Among the final products of alginate digestion by AlyPB1, five major fractions were separately collected for ESI–MS analysis, and five major molecular ion peaks at 351 *m/z* [M−H]^−^, 527 *m/z* [M−H]^−^, 703 *m/z* [M−H]^−^, 879 *m/z* [M−H]^−^ and 1055 *m/z* [M−H]^−^ were individually detected in the corresponding fractions (Fig. 5a–e). These molecular ion peaks could be assigned to unsaturated disaccharide (UDP2), trisaccharide (UDP3), tetrasaccharide (UDP4), pentasaccharide (UDP5) and hexasaccharide (UDP6), respectively, based on the calculation of their molecular masses. The molar ratio of these fractions was calculated to be 23:52:18.6:4:2.4 according to peak area integration of the gel filtration chromatograph. For AlyPB2, the final products had two main molecular ion peaks at 175 *m/z* [M−H]^−^ and 193 *m/z* [M−H]^−^ (Fig. 5f). Previously reported studies have demonstrated that the unsaturated monosaccharides produced by exo-type alginate lyases can be first non-enzymatically converted into 4-deoxy-l-erythro-5-hexoseulose uronic acid (DEH) and then automatically transformed into 2, 4, 5, 6-tetrahydroxytetrahydro-2*H*-pyran-2-carboxylic acid (TPC) [15, 44]. Thus, the main peaks at 175 *m/z* [M−H]^−^ and 193 *m/z* [M−H]^−^ corresponded to unsaturated monosaccharide/DEH and TPC, respectively.

### Sequencing of AlyPB1-produced unsaturated oligosaccharides by ^1^H NMR spectroscopy combined with digestion by AlyPB2

**Fig. 6:**
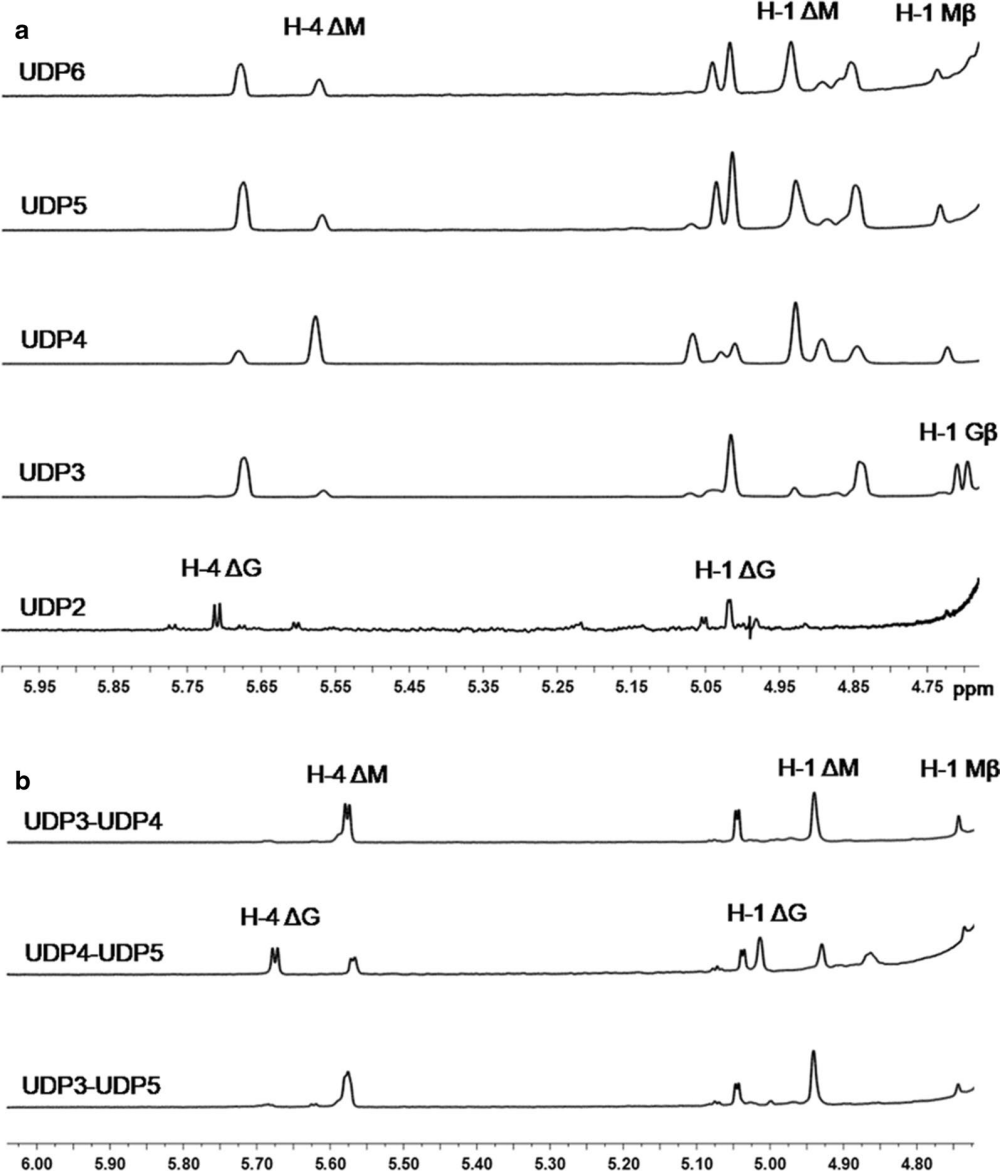
^1^H-NMR analyses of the final oligosaccharide products of alginate digested by AlyPB1. **a** ^1^H-NMR (600 MHz) spectra of the main final products UDP2–UDP6 obtained from sodium alginate digested by AlyPB1. **b** ^1^H-NMR (600 MHz) spectra of the intermediate oligosaccharide products of UDP4 and UDP5 digested by exo-lyase AlyPB2. The intermediate oligosaccharide products UDP3–UDP4, and UDP3–UDP5 and UDP4–UDP5 were individually prepared from the partial digestion of UDP4 and UDP5 by AlyPB2. UDP3–UDP4 represents unsaturated trisaccharides from the final product UDP4; UDP3–UDP5 and UDP4–UDP5 represents unsaturated tri- and tetrasaccharides from the final product UDP5. The most relevant signals are pointed out as follows: the H-4Δ signals at 5.71 or 5.67 ppm indicate that the neighbor to the unsaturated residue (Δ unit) is a G, meaning that ΔG is the first two residues at the non-reducing ends of unsaturated oligosaccharide products. The H-4Δ signals at 5.61 ppm and 5.56 ppm indicate that ΔM is the first two residues at the non-reducing ends. The appearance of a characteristic doublet at 4.71 ppm with ^3^*J*_HH_ = 8.4 Hz corresponds to the β-anomeric proton of a G residue at the reducing end and the single peak occurs at 4.70–4.80 ppm corresponds to β-anomeric proton of a M residue at the reducing end

To further identify the structures of the oligosaccharides produced by AlyPB1, five size-defined oligosaccharide fractions, UDP2–UDP6, were isolated from the final products of alginate digestion by AlyPB1, as described above. Because the chemical shifts of the protons of the Δ unit at the non-reducing end of alginate oligosaccharide are strongly affected by the property of the nearest monosaccharide residues, and the structure of the residue next to the Δ unit can be directly identified based on the H-4 signal of the Δ unit in the ^1^H-NMR spectrum of the unsaturated oligosaccharides [17, 39–42]. In the case of UDP2 (Fig. 6a), the specific signal at 5.71 ppm of H-4 of ΔG was much stronger than that (5.61 ppm) of H-4 of ΔM, indicating that ΔG was the main disaccharide product and that the molar ratio of ΔG to ΔM was approximately 3.4:1.0 (Table 2). Furthermore, the residues at the reducing ends of alginate oligosaccharides can be determined according to the characteristic signals of their anomeric protons because the β-anomeric protons of the *G* and *M* residues at the reducing ends have a characteristic doublet at 4.71 ppm with ^3^*J*_HH_ = 8.4 Hz and a single peak at 4.70–4.80 ppm, respectively [32, 48]. Thus, sequences of UDP3 from the final products can also be directly determined using ^1^H NMR spectroscopy. As shown in Fig. 6a, only the characteristic doublet at 4.71 ppm was detected in the UDP3 sample, indicating that the reducing ends contained only G residues. Moreover, the molar ratio of the non-reducing end ΔG to ΔM was calculated to be 6.7:1 by integrating the areas of the H-4 ΔG (5.67 ppm) and H-4 ΔM (5.56 ppm) signals, suggesting that the UDP3 products contain two unsaturated trisaccharides, ΔGG and ΔMG, at a ratio of 6.7:1 (Table 2).

**Table 2:** The structures and molar fractions of the final unsaturated oligosaccharide products generated by AlyPB1

Final unsaturated oligosaccharide products of AlyPB1	Molar fraction %
UDP2	
ΔG	77
ΔM	23
UDP3	
ΔGG	87
ΔMG	13
UDP4	
ΔMMM	76
ΔGMM	24
UDP5	
ΔGGMM	46.7
ΔMGMM	13.3
ΔGMMM	31.1
ΔMMMM	8.9

By contrast, the sequences of the larger unsaturated oligosaccharide fractions, such as UDP4, UDP5 and UDP6, were difficult to determine through only their ^1^H-NMR spectra; however, their reducing and non-reducing monosaccharide residues can be identified by the method used to identify UDP2 and UDP3. According to the ^1^H-NMR spectra shown in Fig. 6a, structures of UDP4, UDP5 and UDP6 can be preliminarily determined to be ΔGXM and ΔMXM at a molar ratio of 1:3.2; ΔGXXM and ΔMXXM (3.5:1); and ΔGXXXM and ΔMXXXM (1.6:1), in which X represents an unknown residue. Interestingly, the reducing-end residues of all the three larger products, i.e., the UDP4, UDP5 and UDP6 fractions, were M, which has a characteristic single peak at 4.70–4.80 ppm, completely different from the small products (UDP2 and UDP3), which have G as their reducing-end residues (Table 2).

**Fig. 7:**
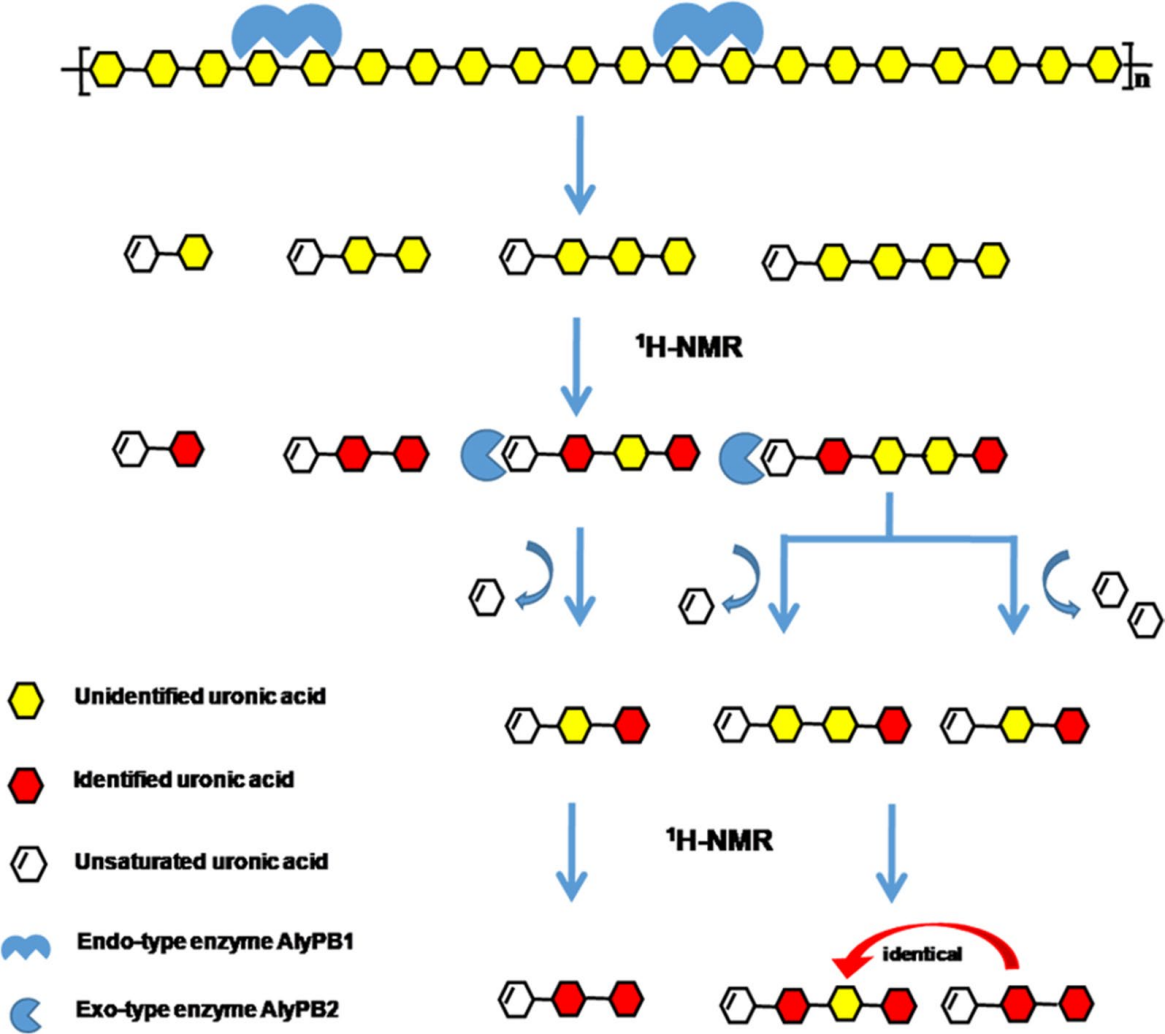
Schematic diagram of the novel method for sequencing alginate oligosaccharides by combining ^1^H NMR spectroscopy and digestion with the exo-lyase AlyPB2

To determine the unknown X residues inside the larger unsaturated alginate oligosaccharides, e.g., the UDP4 and UDP5 fractions, a novel method was established by combining ^1^H NMR spectroscopy with digestion by AlyPB2 (Fig. 7). Since AlyPB2, as a bifunctional exolytic alginate lyase, can sequentially remove monosaccharide residues one by one from the non-reducing end of the alginate chains, the unidentified X residue inside the chain can be exposed as ΔX at the non-reducing end by appropriate partial digestion with AlyPB2, and the corresponding X residue can be easily identified by ^1^H NMR spectroscopy, as mentioned above (Fig. 7). Thus, to identify the internal structures of UDP4 (ΔGXM and ΔMXM) produced by AlyPB1, the unsaturated trisaccharides UDP3–UDP4 (ΔXM and ΔXM) from the reducing ends of UDP4 were prepared by partial digestion with AlyPB2 and analysed by ^1^H NMR spectroscopy. As shown in Fig. 6b, UDP3–UDP4 still showed a single peak at 4.70–4.80 ppm, similar to its parent oligosaccharide UDP4, indicating that the reducing-end M residues were not affected by treatment with AlyPB2. By contrast, a strong signal (5.56 ppm) corresponding to H-4 ΔM but not H-4 ΔG was detected in the ^1^H NMR spectrum of UDP3–UDP4 (Fig. 6b), suggesting that the X residues in both ΔXM and ΔXM are M residues. At this point, it can be concluded that there are two structures, ΔGMM and ΔMMM, at a molar ratio of 1:3.2 in UDP4 produced by AlyPB1 (Table 2).

The structures of final UDP5 product fractions (ΔGXXM and ΔMXXM) produced by AlyPB1 were elucidated by preparing the unsaturated tetrasaccharides UDP4-UDP5 (ΔXXM) and trisaccharides UDP3–UDP5 (ΔXM) at the reducing ends of UDP5 followed by ^1^H NMR analysis, as performed for UDP4 (Figs. 6b, 7). Four structures were identified in the UDP5 fraction, ΔMMMM, ΔMGMM, ΔGMMM and ΔGGMM, which had a molar ratio of ~ 1:1.5:3.5:5.25 (Table 2). However, the structures of UDP6 could not be completely determined due to its relatively low proportion in the final products of alginate by AlyPB1 (Fig. 4a).

### Synergistic effect of AlyPB1 and AlyPB2

**Fig. 8:**
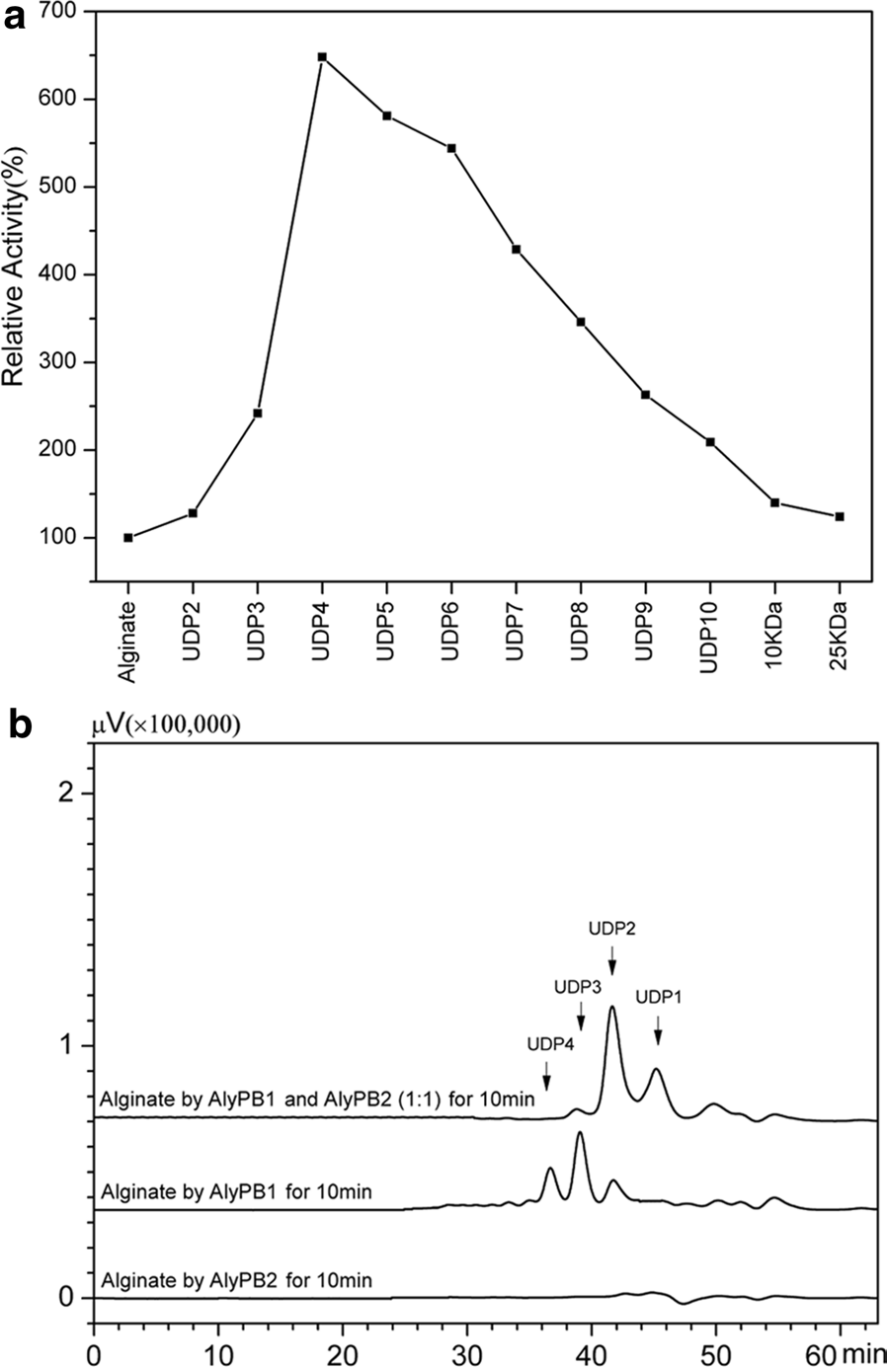
Analysis for synergistic effect of AlyPB1 and AlyPB2. **a** The enzyme activities of exo-lyase AlyPB2 were determined using the alginate with different molecular sizes as substrates. Unsaturated oligosaccharide substrates UDP2–UDP10 were prepared from the partial digestion of alginate by endo-lyase AlyPB1. 10 kDa and 25 kDa alginate polysaccharide were prepared from acid hydrolysis of alginate. Data are shown as the percentage of the activity on alginate (100%). **b** Alginate polysaccharide was treated with a mixture (100 mU) of endo-lyase AlyPB1 and exo-lyase AlyPB2 with an activity ratio of 1:1 at 20 °C for 10 min. By contrast, control was treated with only 100 mU of AlyPB1 or AlyPB2 at the same reaction conditions. The resulting products were analysed by gel filtration as described above at a wavelength of 235 nm. UDP1, UDP2, UDP3, and UDP4 represent unsaturated alginate oligosaccharides as mentioned above

Compared with AlyPB1, AlyPB2 has very low activity when both enzymes act on alginate polysaccharides. Since AlyPB2 is an exolytic lyase, it may prefer to digest small molecular size-defined substrates, such as intermediate oligosaccharide products, generated from the digestion of alginate by AlyPB1. To investigate this possibility, a series of alginate substrates with different molecular sizes was prepared by degradation of alginate with AlyPB1 or an acid. An enzymatic activity assay showed that the activity of AlyPB2 was strongly affected by the size of the substrates (Fig. 8a). Unsaturated tetrasaccharide UDP4 is the optimally sized substrate. Furthermore, the activity of AlyPB2 gradually decreases as the substrate size increases, and its activity towards UDP4 is approximately 6.5 times higher than that towards alginate polysaccharide (Fig. 8a). Notably, the activities of AlyPB2 towards UDP2 and UDP3 were not as high as that towards UDP4 (Fig. 8a), possibly because the former two were too small to bind to the enzyme with high affinity. Nevertheless, UDP2, as the smallest substrate, can be effectively degraded into the unsaturated monosaccharides, indicating that AlyPB1 and AlyPB2 can act together to completely digest alginate into monosaccharide units to facilitate the saccharification of alginate.

To further investigate the cooperative action of AlyPB1 and AlyPB2, alginate polysaccharide was treated with a mixture of these two enzymes (100 mU) with an activity ratio of 1:1 at 20 °C for 10 min. Gel filtration analysis showed that under the given reaction conditions, a small amount of UDP1 was produced when using AlyPB2 only, while the yield of UDP1 was dramatically increased approximately sevenfold when AlyPB1 and AlyPB2 acted on alginate together (Fig. 8b). These results suggest that the digestion of alginate by AlyPB1 strongly promotes the activity of AlyPB2 to release UDP1 from substrates, which is consistent with the finding that AlyPB2 prefers to digest alginate oligosaccharides rather than polysaccharides.

## Discussion

Alginate, as a cost-competitive renewable source for biofuel production, has considerable research prospects [49, 50]. In the saccharification process of alginate, the synergistic effect of various alginate lyases with different substrate specificities and degradation modes plays a vital role [28]. Compared with individual enzyme biocatalysis, multiple enzyme biosystems enable improve utilization of alginate and decrease production costs on the basis of their complementary advantages.

In this study, the endo-lyase AlyPB1 and exo-lyase AlyPB2 were identified from a marine bacterium, *Photobacterium* sp. FC615. AlyPB1 was classified into the PL6 family and had the highest similarity (60.23%) with AlyF, an elucidated alginate lyase in PL6 family [43]. AlyPB2 belongs to the PL15 family and has < 43% sequence identity with most of the previously elucidated exolytic alginate lyases in PL15 family, except for OalA from *Vibrio splendidus* 12B01 [28], which had the highest sequence identity of 93.4% with AlyPB2. AlyPB1 and AlyPB2 are alginate lyases with preferences to polyG and polyM, respectively. AlyPB2 also exhibited obvious activity towards polyG, indicating that AlyPB2 is a bifunctional lyase. Similar to most of the identified enzymes in PL6 family, AlyPB1 has a polyG preference, but it shows much higher enzymatic activity than those of the other identified PL6 family enzymes, even the recently identified AlyF [43]. To date, only five alginate lyases, including AlyPB2, have been classified in the PL15 family, and all of them are exo-type lyases. Notably, the identified exo-type alginate lyases predominantly have a polyM preference, except AlgS6 in PL6 family and AlyA5 in PL7 family [32, 44].

AlyPB1 contains a type I signal peptide composed of 21 amino acids at its N-terminus, while AlyPB2 has no signal peptide, indicating their different subcellular localization in the native bacterium. Therefore, AlyPB1 is a typical secreted enzyme, whereas AlyPB2 is an intercellular enzyme, consistent with their basic enzymatic properties and functions in the alginate degradation. Although the optimal conditions for both enzymes are similar, AlyPB2 is much more sensitive to changes in the reaction conditions, which may be due to its relatively stable intercellular environment. AlyPB2 can be significantly enhanced by reducing agents, indicating that AlyPB2 may active in reducing intracellular environment. By contrast, AlyPB1, as an extracellular enzyme, can adapt to a flexible and dynamic environment. As a result, secreted AlyPB1 degrades alginate polysaccharides outside cells to produce oligosaccharides that can be easily transported into cells for final digestion by AlyPB2.

Exolytic lyases are not only key enzymes for the final digestion and saccharification of alginate but also important tools for the structural analysis of alginate chains. Thus, it is very important to investigate their substrate degradation patterns, such as their substrate preference and degradation direction. In previous studies, researchers mainly focused on substrate preferences but rarely studied the substrate degradation direction of exolytic alginate lyases. In this study, we used a fluorescent labeling method to successfully determine that AlyPB2 removes monosaccharide residues one by one from the non-reducing end of alginate chains. On this basis, we established a novel method for sequencing alginate oligosaccharides by combining ^1^H NMR analysis with exolytic lyase treatment for the first time. Compared to traditional analysis methods based on complex one- and two-dimensional NMR spectroscopy [51, 52], the method described in this paper makes compositional and structural analyses of larger alginate oligosaccharides (≥ UDP4) much easier to perform. The exact structures of the final products produced by many identified endolytic alginate lyases remain to be determined to completely elucidate their action patterns. By contrast, the structures of the main final products of alginate by AlyPB1 were completely determined using the novel method described in this paper. The results showed that UDP2 and UDP3 in the final products were mainly ΔG and ΔGG, whereas all of UDP4 and UDP5 contained a reducing-end MM structure and more than 50% M residues, which suggests that AlyPB1 preferentially digests the G-blocks in alginate chains to produce small ΔG and ΔGG structures but has difficulty cleaving M-rich domains, resulting in larger M-rich oligosaccharides (≥ UDP4) [53, 54].

In most cases, exolytic enzymes show very low enzymatic activity toward polysaccharides compared with those of endolytic enzymes, possibly because polysaccharides are too large to efficiently bind to exolytic enzymes. We are interested in whether exolytic enzymes preferentially degrade oligosaccharides and thus synergistically work with endolytic enzymes. However, this question has rarely been addressed in previous studies on alginate lyases. In this study, we found that the activity of AlyPB2 was substrate size-dependent and that its activity towards alginate oligosaccharides, especially tetrasaccharide chains, was much higher than that towards polysaccharides, similar to the activity of OalA from *Vibrio splendidus* 12B01 [28]. Furthermore, we found that compared to that when AlyPB2 was used alone, the conversion rate of alginate polysaccharides to unsaturated monosaccharides when AlyPB1 and AlyPB2 acted on alginate together was dramatically increased approximately sevenfold over a short time, demonstrating that the synergistic effect of AlyPB1 and AlyPB2 was quite remarkable.

By contrast, a detailed study on the synergistic mechanism of alginate lyases has rarely been reported. Gimpel et al. [31] used 21 different endo-/exo-lyase combinations to investigate the synergistic effect of different pairs, including 7 endo-lyases and 3 exo-lyases. However, they did not investigate the synergistic mechanism of these alginate lyases, while focused on only optimizing the conditions of the enzymatic reactions. Similarly, Wang et al. [30] studied the synergistic action conditions but not mechanism of an endo-lyase and an exo-lyase. Obviously, the synergistic mechanism of endolytic and exolytic alginate lyases has rarely been investigated in past studies. In our study, we elucidated the synergistic mechanism of AlyPB1 and AlyPB2, which is mainly based on the complementarity of their substrate-degrading properties, including their preference for M- and G-blocks and their substrate-size dependence. These findings are very important for the construction of a multi-enzyme system for the saccharification of alginate.

## Conclusions

In conclusion, the identification of the two alginate lyases AlyPB1 and AlyPB2 from *Photobacterium* sp. FC615 provides not only two novel tool-type enzymes but also a good model for studying the synergistic effect of endolytic and exolytic lyases on the saccharification of alginate for biofuel production. Through studies of the enzymatic characteristics, substrate degradation patterns and structures of the final products, the synergistic mechanism of AlyPB1 and AlyPB2 was clearly elucidated, and a novel and simple method for alginate oligosaccharide sequencing using ^1^H NMR spectroscopy and digestion with the exo-lyase AlyPB2 was established for the first time. It is worth emphasizing that compared to traditional methods based on complex one- and two-dimensional NMR spectroscopy, this novel method makes the structural analysis of alginate oligosaccharides larger than trimers easy to understand and carry out, which was proven in this study by the identification of the exact structures of oligosaccharide products generated by AlyPB1. In fact, the sequences of complex alginate oligosaccharides such as tetra- and pentasaccharides produced by most alginate lyases have rarely been sequenced in past studies, severely hindering further studies on related enzymes and their products. We believe that our method will be a powerful solution to this important issue, especially for more complex alginate oligosaccharides obtained from enzymatic, chemical or physical methods. In summary, this research will provide important theoretical and technical support for synergistic degradation as well as structure–function studies of alginate based on alginate lyases.

## Data Availability

Not applicable.

## Abbreviations

Δ: 4-Deoxy-l-erythro-5-hexoseulose uronic acid
TPC: 2,4,5,6-Tetrahydroxytetrahydro-2*H*-pyran-2-carboxylic acid
DP: Degree of polymerization
G: Guluronate
M: Mannuronate
PolyG: Polyguluronate
PolyM: Polymannuronate
SDS-PAGE: Sodium dodecyl sulfate polyacrylamide gel electrophoresis
HPLC: High-performance liquid chromatography
NMR: Nuclear magnetic resonance
PL family: Polysaccharide lyase family
UDP1: Unsaturated monosaccharide
UDP2: Unsaturated disaccharide
UDP3: Unsaturated trisaccharide
UDP4: Unsaturated tetrasaccharide
UDP5: Unsaturated pentasaccharide
UDP6: Unsaturated hexasaccharide
DP5: Saturated alginate pentasaccharide
